# The GPX3‐VCAM1 Axis Gates Pro‐Fibrotic Tubule Cell Fate in Hyperuricemic Nephropathy

**DOI:** 10.1002/advs.77676

**Published:** 2026-09-09

**Authors:** Yunfei Qi, Qiang Zhao, Yue Lve, Yaming Yuan, Tingting Zhou, Yue Yuan, Dexi Wu, Yifan Tang, Yan Liu, Zhigang Li, Chunping Jiang, Qiurong Ding

**Affiliations:** ^1^ Shanghai Institute of Nutrition and Health Chinese Academy of Sciences University of Chinese Academy of Sciences Shanghai China; ^2^ Department of Cardiology Shanghai Changzheng Hospital Naval Medical University Shanghai China; ^3^ Department of Urology Xinhua Hospital Affiliated to Shanghai Jiaotong University School of Medicine Shanghai China; ^4^ Division of Hepatobiliary and Transplantation Surgery Department of General Surgery, Nanjing Drum Tower Hospital the Affiliated Hospital of Medical School Nanjing University Nanjing China; ^5^ Jinan Microecological Biomedicine Shandong Laboratory Jinan China; ^6^ Department of Hepatobiliary and Pancreatic Surgery The Second Affiliated Hospital of Fujian Medical University Quanzhou China; ^7^ Renhuai People's Hospital Renhuai Guizhou Province China; ^8^ Shanghai Key Laboratory of Reproductive Medicine Shanghai China; ^9^ Hubei Key Laboratory of Selenium Resource Research and Biological Application Hubei Minzu University Enshi China

**Keywords:** GPX3, hyperuricemia, pro‐fibrotic proximal tubule cells, renal fibrosis, selenium, VCAM1

## Abstract

Hyperuricemia is a major driver of chronic kidney disease, yet the underlying intrarenal mechanisms remain unclear. Here, we establish a genetically defined, multi‐hit mouse model that recapitulates the sustained hyperuricemia, metabolic dysregulation, and progressive fibrosis of human disease. Using this model, we demonstrate that soluble uric acid activates the renal NLRP3 inflammasome and that its inhibition attenuates kidney injury. Single‐nucleus RNA sequencing revealed a distinct pro‐inflammatory and pro‐fibrotic proximal tubule cell population (Profib PT) marked by high VCAM1 expression, which is conserved in human kidney disease. Direct VCAM1 neutralization with antibodies attenuated renal fibrosis, validating its functional role. Mechanistically, we identify glutathione peroxidase 3 (GPX3) as a master regulator restraining precursor cell differentiation into Profib PT cells. GPX3 expression is suppressed in hyperuricemia, licensing this pathogenic transition; conversely, dietary selenium supplementation restored GPX3 activity, blocked Profib PT differentiation and VCAM1‐dependent macrophage adhesion, and ameliorated renal fibrosis. Our findings define the GPX3‐VCAM1 axis as a central regulatory node in hyperuricemic nephropathy and identify nutritional selenium repletion as a novel therapeutic strategy to intercept fibrosis.

## Graphical abstract

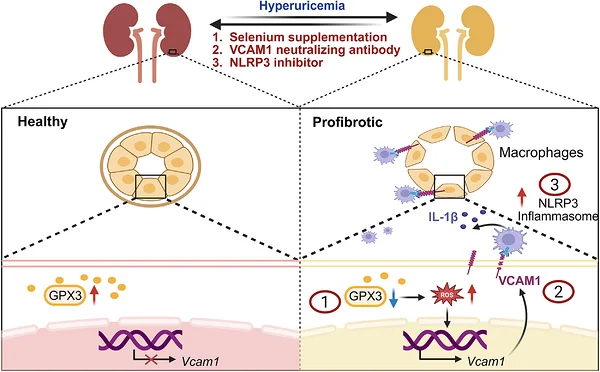

## 1 Introduction

Hyperuricemia is a highly prevalent metabolic disorder and a well‐established risk factor for gout. Beyond its articular manifestations, a growing body of clinical and epidemiological evidence links elevated serum uric acid to the development and progression of chronic kidney disease (CKD), independent of traditional risk factors [1, 2, 3, 4, 5, 6, 7, 8]. This condition, termed hyperuricemic nephropathy or urate nephropathy, is characterized by tubulointerstitial inflammation, fibrosis, and progressive loss of renal function. Despite this strong association, it remains debated whether asymptomatic hyperuricemia confers similar risk for CKD [2, 9, 10], and the specific intrarenal mechanisms by which soluble uric acid drives kidney injury remain incompletely understood. This knowledge gap hinders the development of targeted therapies beyond urate‐lowering agents.

Current mechanistic understanding has largely focused on the activation of the NLRP3 (NOD‐, LRR‐, and pyrin domain‐containing protein 3) inflammasome by urate crystals within joints, a cornerstone of gout pathophysiology, which can drive acute and chronic renal inflammation [11, 12]. The role of soluble uric acid is more controversial. While some in vitro and in vivo studies suggest it can also activate NLRP3 [13], Toll‐like receptor 4 (TLR4) [14, 15], and other inflammatory pathways [16, 17], possibly via inducing oxidative stress, endothelial dysfunction, or disruption of sodium transport [18, 19, 20, 21, 22], the precise mechanisms are not fully resolved. Nonetheless, both crystalline and soluble uric acid are strongly linked to oxidative stress—a key pathogenic mediator—through increased xanthine oxidase activity, nicotinamide adenine dinucleotide phosphate (NADPH) oxidase stimulation, and mitochondrial dysfunction [12].

The renal proximal tubule, a primary site of urate handling and oxidative stress, is critically involved in the pathogenesis of various CKD etiologies. Recent single‐cell studies have identified distinct profibrotic proximal tubule cell (PTC) subpopulations that emerge following acute kidney injury (AKI) and drive the transition to CKD. For example, Failed‐Repair PTCs (FR‐PTCs) persist after ischemia‐reperfusion injury, expressing high levels of pro‐inflammatory and pro‐fibrotic genes [23, 24, 25]; and a subset of kidney proximal tubule cells present with high expression of cytokines and chemokines associated with immune cell recruitment in animals subjected to unilateral ureter obstruction (UUO) surgery [26]. However, the specific PTC heterogeneity and the injury‐specific subpopulations that orchestrate hyperuricemia‐induced fibrosis—a prototypical chronic process—have not been explored. This lack of resolution at the single‐cell level represents a significant barrier to elucidating cell‐specific disease mechanisms.

Furthermore, the development of reliable preclinical models that faithfully recapitulate human hyperuricemic nephropathy is paramount. Many existing hyperuricemia models rely on pharmacologic (e.g., potassium oxonate treatment) or genetic inhibition of uricase (e.g., whole‐body knockout of *Uox*), or extreme dietary manipulations (e.g., high‐fructose/high‐purine diet), which often fail to produce sustained, human‐relevant pathology or introduce confounding systemic effects, including drug toxicity, homozygous embryonic lethality, and widespread systemic disturbances [27]. Given that human hyperuricemia is a complex disease influenced by genes and environmental factors, there is a pressing need for a stable, physiologically relevant model to systematically dissect the renal‐specific sequelae of chronic hyperuricemia.

In this study, we address these critical gaps. First, we establish and validate a novel, robust mouse model of hyperuricemia using liver‐specific CRISPR‐Cas9‐mediated knockout of urate oxidase (*Uox*), combined with moderate dietary and hypoxanthine challenges. Using this model, we confirm the pathogenic role of the renal NLRP3 inflammasome and demonstrate the therapeutic efficacy of its inhibition. Furthermore, we employ single‐nucleus RNA sequencing (snRNA‐seq) to deconvolute the renal cellular landscape. This approach led to the discovery of a previously unrecognized pro‐fibrotic proximal tubule cell subpopulation (Profib PT) that emerges specifically in hyperuricemic kidneys and expresses a conserved marker, VCAM1. We further delineate the cellular origin of these Profib PT cells, identifying glutathione peroxidase 3 (GPX3) as a master regulator whose loss, driven by oxidative stress, licenses their differentiation. Finally, we translate this mechanistic insight into a novel therapeutic strategy, demonstrating that dietary selenium supplementation restores GPX3 activity, suppresses the Profib PT phenotype, and effectively attenuates renal inflammation and fibrosis.

In conclusion, our work provides a validated experimental platform for hyperuricemic nephropathy and unveils the GPX3‐VCAM1 axis in proximal tubules as a central regulatory node and a promising therapeutic target for a condition with limited treatment options.

## 2 Results

### 2.1 A Novel Hyperuricemia Model Recapitulates Human Renal Pathology

**FIGURE 1:**
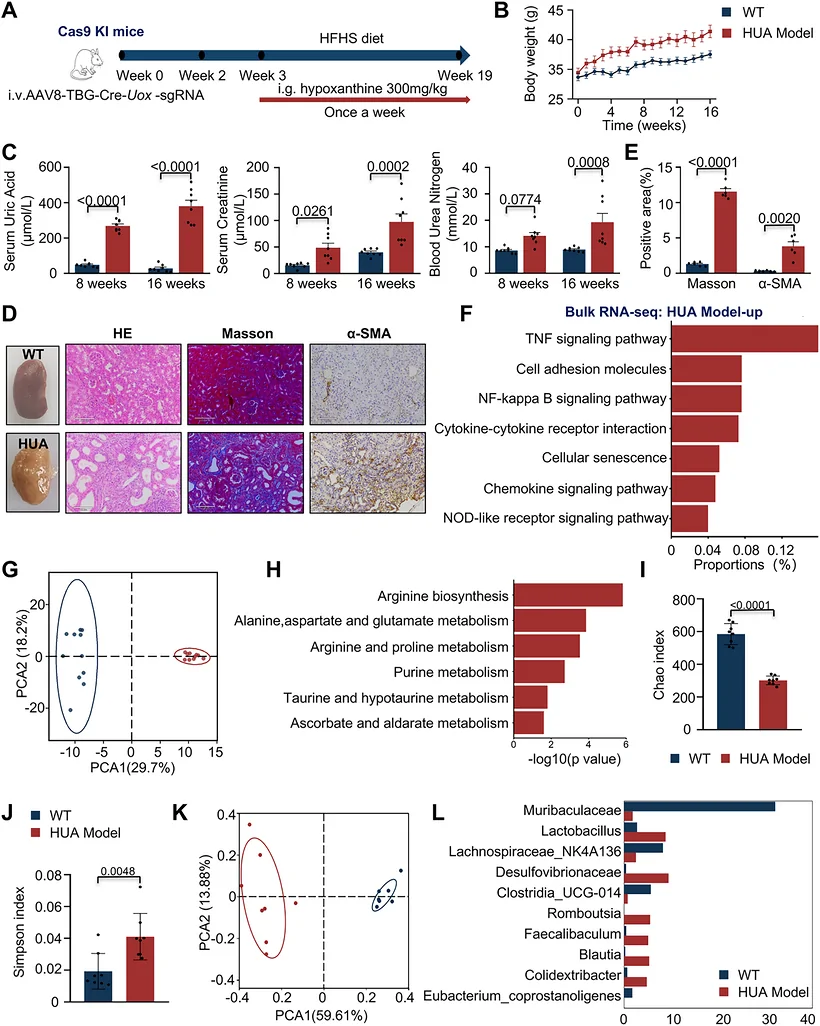
Establishment and comprehensive characterization of a novel hyperuricemia mouse model. (A) Schematic overview of the experimental design. The Cas9 knock‐in mice were injected intravenously with AAV8‐TBG‐*Uox*‐sgRNA at week 0, followed by a high‐fat/high‐sucrose (HFHS) diet. Hypoxanthine (300 mg/kg) was administered intragastrically once per week from week 3 to week 19 to induce hyperuricemia. (B) Body weight changes in wild‐type (WT) and HUA model mice during the experimental period (*n* = 8). (C) Serum biochemical parameters, including serum uric acid, creatinine, and blood urea nitrogen (BUN), measured at 8 and 16 weeks (*n* = 8). Statistical significance was determined by two‐way ANOVA followed by Sidak's multiple comparisons test. (D) Representative gross kidney images and histological analyses, including hematoxylin and eosin (H&E) staining, Masson's trichrome staining, and α‐smooth muscle actin (α‐SMA) immunohistochemistry, showing renal structural damage and fibrosis in HUA mice. Scale bar: 110 µm. (E) Quantitative analysis of Masson and α‐SMA staining positive areas (%) in kidney sections (*n* = 6). Statistical significance was determined by an unpaired two‐tailed t test. (F) KEGG pathway enrichment analysis of upregulated genes identified by bulk RNA sequencing of kidney tissues from HUA model mice, revealing activation of inflammatory and fibrosis‐related signaling pathways. (G) Principal component analysis (PCA) score plot of serum metabolomic profiles showing a clear separation between WT and HUA model mice (*n* = 8). (H) KEGG pathway enrichment analysis of significantly altered serum metabolites, highlighting dysregulated metabolic pathways in HUA mice (*n* = 8). (I, J) Alpha diversity analysis of gut microbiota based on the Chao index (I) and Simpson index (J) (*n* = 8). Statistical significance was determined by an unpaired two‐tailed *t*‐test. (K) PCA of gut microbiota composition demonstrating distinct microbial community structures between WT and HUA mice (*n* = 8). (L) Relative abundance of representative bacterial taxa at the genus/family level in WT and HUA model mice (*n* = 8).

Given the multifactorial etiology of human hyperuricemia—which stems from genetic predisposition (e.g., the evolutionary loss of uricase) and modifiable lifestyle factors (e.g., diets high in fructose and purines)—we developed a combinatorial mouse model to recapitulate these pathogenic drivers. The integrated approach was designed to move beyond single‐factor models by combining genetic susceptibility with chronic dietary and metabolic challenges, thereby establishing a stable, clinically relevant platform for studying hyperuricemic nephropathy. To this end, we generated a hyperuricemia (HUA) model in which liver‐specific knockout of urate oxidase (*Uox*) was achieved in Cas9 knock‐in mice via AAV8‐TBG‐*Uox*‐sgRNA delivery, mimicking the genetic basis of human hyperuricemia. These mice were then subjected to a high‐fat/high‐sucrose (HFHS) diet to simulate a Western dietary pattern, supplemented with weekly intragastric administration of hypoxanthine (300 mg/kg) to impose a purine load reflective of episodic high intake (Figure 1A). Following sgRNA screening, the knockout efficiency of hepatic *Uox* was validated at both the mRNA and protein levels, which showed marked reductions in HUA model mice (Figure S1A–D).

Compared to wild‐type (WT) controls, HUA mice exhibited progressive weight gain (Figure 1B) and sustained hyperuricemia, with serum urate levels rising to ∼250 µmol/L at 8 weeks and to ∼400 µmol/L at 16 weeks (Figure 1C). By 16 weeks, elevated serum creatinine and BUN confirmed renal impairment (Figure 1C). Kidneys from HUA mice showed gross morphological changes, histopathological injury, increased collagen deposition, and enhanced α‐SMA expression, indicating fibrosis (Figure 1D,E). Notably, polarized light microscopy revealed no birefringence or visible urate crystal deposition within the renal tubular lumens (Figure S1E). Taken together, these results demonstrate that soluble uric acid, independent of crystal deposition, is capable of inducing renal injury and fibrosis in this model. Bulk RNA sequencing of kidney tissue revealed significant upregulation of pathways involved in inflammation (e.g., TNF, NF‐κB, NLR signaling) and fibrosis (Figure 1F), collectively demonstrating successful modeling of hyperuricemic nephropathy.

Notably, neither hepatic *Uox* knockout alone nor the dietary/hypoxanthine challenge in WT mice induced sustained hyperuricemia or nephropathy (Figure S1F,G). Furthermore, female mice exhibited an attenuated hyperuricemic response compared to males, with serum uric acid levels significantly elevated but remaining far below male levels, while serum creatinine and BUN showed no intergroup differences (Figure S2A,B). These findings suggest that females are relatively resistant to hyperuricemia and largely protected from kidney injury under the same protocol, consistent with established sex differences in human hyperuricemia [28].

Hyperuricemia is usually associated with metabolic reprogramming. Studies with human populations demonstrated significantly increased metabolites associated with arginine and proline metabolism pathways in serum in hyperuricemia patients [29]. Interestingly, untargeted serum metabolomic analysis in these animals also revealed distinct metabolic profiles between WT and HUA mice (Figure 1G). Pathway enrichment of differentially abundant metabolites highlighted significant perturbations in arginine biosynthesis, alanine/aspartate/glutamate metabolism, arginine and proline metabolism, and purine metabolism (Figure 1H), mirroring metabolic reprogramming observed in hyperuricemic patients.

Finally, consistent with observations in hyperuricemic patients [30, 31, 32], 16S rRNA sequencing revealed significant gut microbiota dysbiosis in HUA mice. This was characterized by reduced alpha diversity (Chao and Simpson indices; Figure 1I,J), distinct beta‐diversity clustering (Figure 1K), and alterations in specific bacterial taxa. Notably, we observed changes in the relative abundance of *Muribaculaceae*, *Lactobacillus*, and *Desulfovibrionaceae*—genera whose dysregulation is frequently associated with human hyperuricemia [30, 31, 32], underscoring the clinical relevance of our model (Figure 1L).

In summary, this HUA model recapitulates sustained hyperuricemia, progressive nephropathy, systemic metabolic disturbances, and gut dysbiosis, providing a robust and clinically relevant platform for mechanistic and therapeutic discovery.

### 2.2 NLRP3 Inflammasome Signaling Drives Renal Fibrosis in Hyperuricemia

**FIGURE 2:**
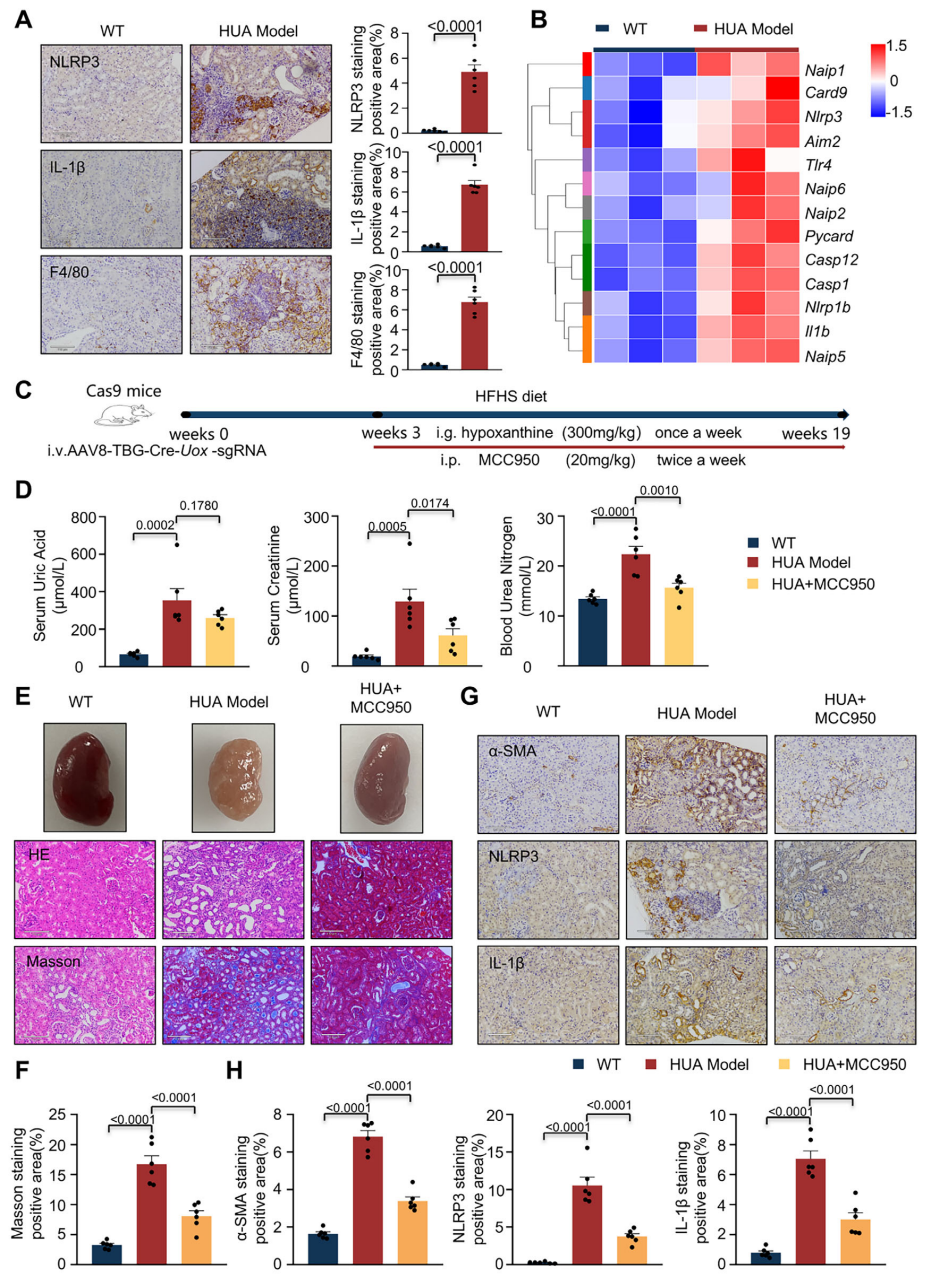
Renal NLRP3 inflammasome signaling contributes to hyperuricemic nephropathy. (A) Representative immunohistochemical staining and corresponding quantification of NLRP3, IL‐1β, and the macrophage marker F4/80 in kidney sections from wild‐type (WT) and hyperuricemia (HUA) model mice, showing enhanced inflammasome activation and macrophage infiltration in HUA kidneys (*n* = 6). Scale bar: 110 µm. Statistical significance was determined by an unpaired two‐tailed *t*‐test. (B) Heatmap illustrating the relative expression of inflammasome‐related genes in kidneys from WT and HUA model mice, including *Nlrp3*, *Aim2*, *Pycard*, *Casp1*, *Il1b*, and related pattern recognition receptors, indicating broad activation of inflammasome‐associated pathways in HUA kidneys. (C) Schematic of the experimental design. Cas9 knock‐in mice were injected intravenously with AAV8‐TBG‐*Uox*‐sgRNA and fed a high‐fat/high‐sucrose (HFHS) diet with weekly intragastric hypoxanthine administration (300 mg/kg). The NLRP3 inhibitor MCC950 (20 mg/kg) was administered intraperitoneally twice per week starting at week 3. (D) Serum biochemical analyses showing serum uric acid, serum creatinine, and blood urea nitrogen (BUN) levels in WT, HUA model, and HUA mice treated with MCC950. MCC950 treatment significantly reduced renal dysfunction markers and partially lowered serum uric acid levels (*n* = 6). Statistical differences among groups were analyzed using ordinary one‐way ANOVA followed by Tukey's post hoc test (*p* = 0.0003 for serum uric acid; *p* = 0.0008 for serum creatinine; *p* = 0.0385 for BUN). (E) Representative gross kidney morphology and renal histology assessed by hematoxylin and eosin (H&E) and Masson's trichrome staining. HUA mice exhibited marked renal structural damage and fibrosis, which were substantially alleviated by MCC950 treatment. Scale bar: 110 µm. (F) Quantitative analysis of the Masson staining positive area (%) in renal tissues (*n* = 6). Statistical differences among groups were analyzed using ordinary one‐way ANOVA followed by Tukey's post hoc test (p < 0.0001). (G) Immunohistochemical staining of α‐smooth muscle actin (α‐SMA), NLRP3, and IL‐1β in kidney sections, demonstrating that MCC950 treatment attenuated inflammasome activation and fibrotic responses in HUA kidneys. Scale bar: 110 µm. (H) Quantitative analysis of IHC positive areas (%) for α‐SMA, NLRP3, and IL‐1β staining in renal sections (n = 6). Statistical differences among groups were analyzed using ordinary one‐way ANOVA followed by Tukey's post hoc test (*p* < 0.0001 for α‐SMA; *p* < 0.0001 for NLRP3; *p* < 0.0001 for IL‐1β).

While NLRP3 inflammasome activation is an established mechanism in gout, its role in hyperuricemia‐induced kidney injury is less clear. We therefore investigated NLRP3 pathway activation in our HUA model. Immunohistochemistry revealed markedly increased renal expression of NLRP3 and IL‐1β, alongside enhanced F4/80^+^ macrophage infiltration, indicating inflammasome‐driven renal inflammation (Figure 2A). Transcriptomic analysis confirmed coordinated upregulation of inflammasome‐related genes (e.g., *Nlrp3*, *Casp1*, *Il1b*) and associated pattern recognition receptors in HUA kidneys (Figure 2B), demonstrating broad activation of this pathway.

To define its functional contribution, we treated HUA mice with the selective NLRP3 inhibitor MCC950 [33, 34] (Figure 2C). MCC950 administration significantly improved renal function, reducing serum creatinine and BUN levels with no significant effect on serum uric acid levels (Figure 2D). Histologically, MCC950 treatment markedly attenuated hyperuricemia‐induced renal structural damage and interstitial fibrosis (Figure 2E,F). Further immunohistochemistry confirmed that NLRP3 inhibition reduced the expression of NLRP3, IL‐1β, and the fibrosis marker α‐SMA (Figure 2G,H).

Collectively, these results demonstrate that soluble uric acid activates renal NLRP3 inflammasome signaling, which is a key driver of inflammation and fibrosis in hyperuricemic nephropathy, and that its pharmacological inhibition is renoprotective.

#### 2.2.1 Identification of a Pro‐Fibrotic Proximal Tubule Cell (Profib PT)

**FIGURE 3:**
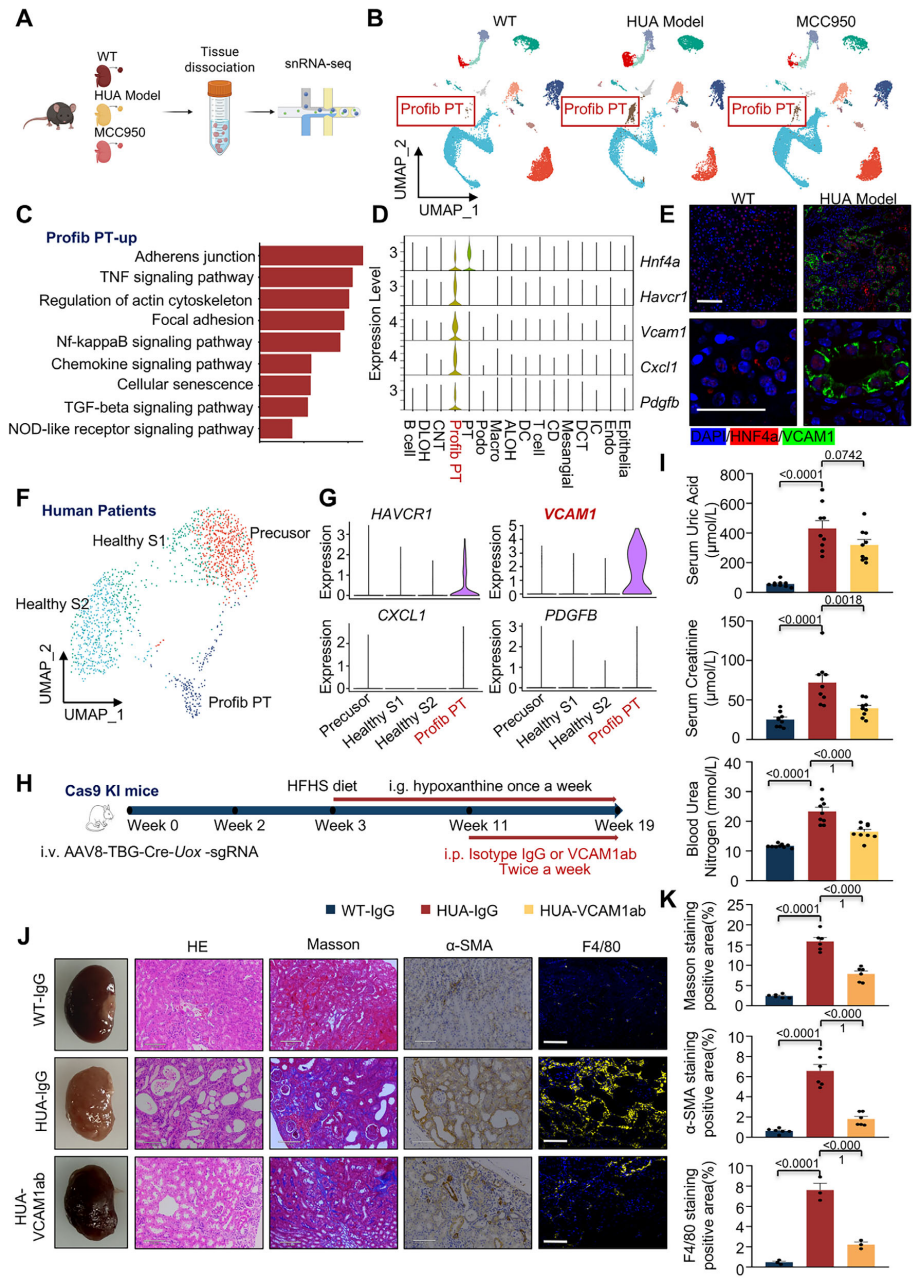
Single‐nucleus RNA sequencing identifies a pro‐fibrotic proximal tubular epithelial cell population in hyperuricemic nephropathy. (A) Schematic of the experimental workflow for kidney single‐nucleus RNA sequencing (snRNA‐seq) in wild‐type (WT), hyperuricemia (HUA) model, and HUA mice treated with the NLRP3 inhibitor MCC950. (B) UMAP visualization of renal cell populations from WT, HUA model, and HUA+MCC950 mice. A distinct cluster of proximal tubular epithelial cells enriched in HUA kidneys was identified and designated as pro‐fibrotic proximal tubule cells (Profib PT). (C) Gene set enrichment analysis of Profib PT cells showing upregulation of pathways associated with cell adhesion, cytoskeletal remodeling, inflammatory signaling (TNF, NF‐κB, chemokine signaling), cellular senescence, and TGF‐β signaling. (D) Violin plots showing expression of injury‐associated and pro‐inflammatory/pro‐fibrotic genes across major renal cell types, highlighting selective enrichment of *Havcr1*, *Vcam1*, *Cxcl1*, and *Pdgfb* in Profib PT cells. (E) Representative immunofluorescence staining of kidney sections from WT and HUA mice demonstrating colocalization of the proximal tubule epithelial marker HNF4A with VCAM1, confirming the presence of Profib PT cells in vivo. Scale bar: 50 µm (top row); 10 µm (bottom row). (F) UMAP projection of proximal tubular epithelial cells from human early‐stage diabetic kidney disease datasets, identifying a Profib PT–like population characterized by elevated expression of *HAVCR1* and *VCAM1*. (G) Violin plots showing expression of *HAVCR1*, *VCAM1*, *CXCL1*, and *PDGFB* across human proximal tubule subpopulations, highlighting conserved enrichment of VCAM1 in Profib PT cells between human and mouse kidneys. (H) Experimental design for VCAM1 neutralizing antibody treatment in HUA model mice. (I) Serum biochemical analyses showing that VCAM1 neutralization significantly reduced serum uric acid, serum creatinine, and blood urea nitrogen (BUN) levels in HUA mice (*n* = 9). Statistical differences among groups were analyzed using ordinary one‐way ANOVA followed by Tukey's post hoc test (*p* < 0.0001 for serum uric acid; *p* < 0.0001 for serum creatinine; *p* < 0.0001 for BUN). (J) Representative gross kidney morphology, histological staining (H&E and Masson's trichrome), and immunostaining for α‐SMA and F4/80. VCAM1 antibody treatment markedly attenuated renal fibrosis and macrophage infiltration in HUA mice. Scale bar: 110 µm for H&E, Masson's trichrome staining, and α‐SMA staining; 50 µm for F4/80 staining. (K) Quantitative analysis of Masson, α‐SMA (*n* = 6) and F4/80 (*n* = 3) staining positive areas (%) in the respective groups. Statistical differences among groups were analyzed using ordinary one‐way ANOVA followed by Tukey's post hoc test (*p* < 0.0001 for Masson; *p* < 0.0001 for α‐SMA; *p* < 0.0001 for F4/80).

Having established the role of the NLRP3 inflammasome, we next aimed to identify the specific renal cell populations driving pathology. We performed single‐nucleus RNA sequencing (snRNA‐seq) on kidneys from wild‐type (WT), HUA, and MCC950‐treated HUA mice (Figure 3A; cell annotation in Figure S3A–C).

Unsupervised clustering revealed a distinct subset of proximal tubular epithelial cells (PTECs) that was markedly expanded in the HUA kidney but largely absent in WT control and reduced by MCC950 treatment (Figure 3B). We designated this injury‐associated population as pro‐fibrotic PT cells (Profib PT). Subclustering of PTECs identified five states: precursor cells, three healthy PT subsets (S1‐S3), and the Profib PT cluster (Figure S3D). Gene set enrichment analysis showed Profib PT cells were enriched for pathways involved in adhesion, cytoskeletal regulation, and inflammatory/fibrotic signaling (TNF/NF‐κB, TGF‐β) (Figure 3C). Profib PT cells selectively expressed high levels of injury and inflammatory mediators, including *Havcr1*, *Vcam1*, *Cxcl1*, and *Pdgfb* (Figure 3D). Notably, the elevated expression of *Pdgfb* in this population suggests that these cells may secrete paracrine mediators capable of promoting fibroblast activation. In addition, this population preferentially expressed several fibrosis‐associated and extracellular matrix‐related genes, including *Fn1*, *Col4a1*, *Col4a2*, and *Spp1* (Figure S3E). Immunofluorescence staining further confirmed the in vivo emergence of VCAM1**^+^** HNF4A**^+^** proximal tubular epithelial cells in HUA kidneys (Figure 3E; Figure S3F). Importantly, VCAM1^+^ cells also expressed LRP2 (Megalin), a well‐established marker of proximal tubular cells, thereby confirming their proximal tubular identity (Figure S3G). Moreover, collagen I, a canonical marker of fibrotic extracellular matrix deposition, prominently accumulated in the interstitial regions surrounding VCAM1^+^ proximal tubular cells in HUA kidneys (Figure S3G). Collectively, the localization of VCAM1^+^ proximal tubular cells within collagen‐rich fibrotic regions, together with their expression of profibrotic mediators and fibrosis‐associated genes, provides additional support for their designation as a profibrotic proximal tubular cell state.

To assess translational relevance, we analyzed published human early diabetic kidney disease (DKD) snRNA‐seq data [35]. A conserved Profib PT‐like population was also identified in human kidneys (Figure 3F; Figure S4A), enriched for adhesion, ECM, and inflammation pathways (Figure S4B). Among Profib PT‐associated markers, VCAM1 showed consistent enrichment across species (Figure 3G). VCAM1 (vascular cell adhesion molecule 1) is an immunoglobulin superfamily adhesion molecule predominantly expressed on endothelial cells, where it plays established roles in chronic inflammation and tissue fibrosis via immune modulation [36, 37, 38, 39]. Notably, VCAM1 signaling can directly influence macrophage/monocyte phenotypes, promoting a pro‐fibrotic state [37, 38, 39]. However, its function in other cell types, including PTECs, remains unclear.

To further delineate the functional role of the VCAM1^+^ PTECs population, we treated HUA mice with a VCAM1‐neutralizing antibody (Figure 3H). VCAM1 blockade significantly improved serum creatinine and BUN levels with no significant effect on serum uric acid levels (Figure 3I). Bulk RNA‐seq of kidneys revealed downregulation of ECM, inflammation, and adhesion pathways post‐treatment (Figure S4C). Histologically, VCAM1 inhibition markedly attenuated renal fibrosis and macrophage infiltration (Figure 3J,K).

In summary, we identified Profib PT as a novel, pathogenic PTEC subpopulation that drives hyperuricemic nephropathy, with VCAM1 as a conserved and therapeutically targetable marker.

### 2.3 GPX3 Restrains Profib PT Differentiation via Redox Regulation

**FIGURE 4:**
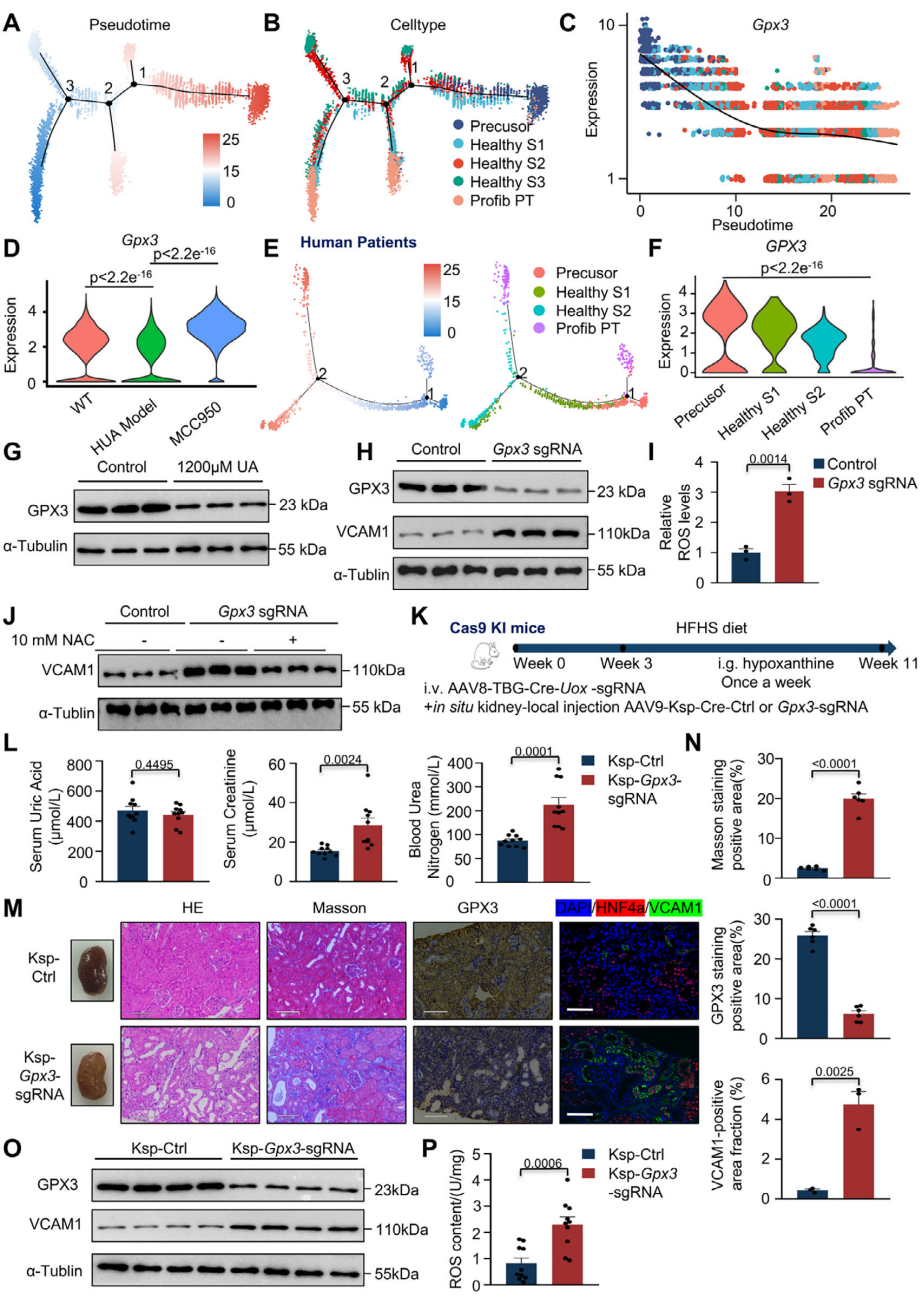
Loss of GPX3 drives the development of VCAM1^+^ Profib PT cells. (A, B) Pseudotime trajectory of proximal tubular epithelial cells inferred from single‐nucleus transcriptomic data. The trajectory is colored by pseudotime values in (A) to depict the transcriptional continuum, and by proximal tubule cell subtypes in (B), including homeostatic PT subsets (Healthy S1–S3) and pro‐fibrotic proximal tubule cells (Profib PT), suggesting that Profib PT cells localize at the late stage of this pseudotemporal ordering. (C) Scatter plot showing dynamic changes in *Gpx3* expression along pseudotime, revealing a progressive decline during differentiation toward the Profib PT state. (D) Violin plots of *Gpx3* expression in kidneys from WT, HUA model, and MCC950‐treated mice, showing reduced *Gpx3* expression in HUA kidneys and partial restoration following NLRP3 inhibition. Statistical significance was determined by the Wilcoxon rank‐sum test. (E) Pseudotime trajectory analysis of proximal tubular epithelial cells from human early‐stage diabetic kidney disease samples suggested a transcriptional progression from homeostatic toward injury‐associated states. (F) Violin plots showing *GPX3* expression across human PT subpopulations, with the lowest expression observed in Profib PT cells. Statistical significance was determined by the Wilcoxon rank‐sum test. (G) Western blot analysis of GPX3 expression in HK2 cells treated with 1200 µM uric acid (UA) for 5 days, showing UA‐induced downregulation of GPX3 (*n* = 3). (H) Western blot analysis of GPX3 and VCAM1 following *Gpx3* knockout in HK2 cells, demonstrating increased VCAM1 expression upon GPX3 loss (*n* = 3). (I) Quantification of intracellular reactive oxygen species (ROS) levels following *Gpx3* knockout (*n* = 3). Statistical significance was determined by an unpaired two‐tailed *t*‐test. (J) Western blot analysis showing that antioxidant treatment with N‐acetylcysteine (NAC) attenuates VCAM1 upregulation induced by Gpx3 knockout (*n* = 3). (K) Schematic of the in vivo experimental design for tubule epithelium–specific *Gpx3* deletion achieved by kidney‐local injection of AAV9‐Ksp‐*Gpx3*‐sgRNA in Cas9 knock‐in mice with established hyperuricemia. (L) Serum creatinine and blood urea nitrogen (BUN) levels in mice with proximal tubule–specific *Gpx3* knockout, indicating aggravated renal dysfunction (n = 10). Statistical significance was determined by an unpaired two‐tailed *t*‐test. (M) Representative gross kidney morphology, H&E staining, Masson's trichrome staining, and immunostaining for GPX3 (Scale bar: 100 µm) and immunofluorescence staining for HNF4α with VCAM1 (Scale bar: 50 µm), showing enhanced renal fibrosis and VCAM1 expression following knockdown. (N) Quantitative analysis of Masson, α‐SMA (*n* = 6), and VCAM1 (*n* = 3) staining positive areas (%) in the respective groups. Statistical significance was determined by an unpaired two‐tailed *t*‐test. (O) Western blot analysis of GPX3 and VCAM1 expression in kidney tissues from control and proximal tubule–specific *Gpx3* knockout mice (*n* = 4). (P) Quantification of renal ROS content, showing increased oxidative stress upon *Gpx*3 loss (*n* = 10). Statistical significance was determined by an unpaired two‐tailed *t*‐test.

We next investigated the origin and regulation of Profib PT cells. Pseudotime trajectory analysis of PTECs suggested a transcriptional progression from a homeostatic state toward injury‐associated states. Profib PT cells fell at the extreme end of this pseudotemporal ordering, correlating with the acquisition of a profibrotic transcriptional signature (Figure 4A,B).

Analysis of gene expression along this trajectory showed a progressive decline in *Gpx3* expression as cells differentiated toward the Profib PT state (Figure 4C; Figure S5A). Consistently, *Gpx3* expression was significantly reduced in HUA kidneys and partially restored by MCC950 treatment (Figure 4D). This precursor‐to‐injury trajectory and GPX3 downregulation were conserved in human diabetic kidney disease, where Profib PT‐like cells exhibited the lowest GPX3 levels among PTEC subsets [35] (Figure 4E,F; Figure S5B).

GPX3 (Glutathione Peroxidase 3) is an extracellular selenoprotein predominantly synthesized by the kidney proximal tubule epithelium [40, 41]. Previous studies have indicated GPX3 as a potent protective factor against kidney injury, primarily attributed to its ability to mitigate oxidative stress [42, 43]. We next further investigated the function of GPX3 in HUA models. In vitro treatment of human proximal tubular epithelial (HK2) cells with uric acid for 5 days significantly reduced GPX3 expression (Figure 4G, Figure S5C). GPX3 knockout in HK2 cells in vitro increased reactive oxygen species (ROS) and upregulated VCAM1 expression, effects rescued by the antioxidant N‐acetylcysteine (NAC) (Figure 4H–J; Figure S5D,E). Similarly, uric acid treatment induced VCAM1, which was attenuated by NAC (Figure S5F), confirming that GPX3 restrains VCAM1 via redox regulation.

To validate this axis in vivo, we next generated a hyperuricemia model with tubular epithelium‐specific *Gpx3* deletion via kidney‐localized AAV9‐Ksp‐*Gpx3*‐sgRNA delivery (Figure 4K). To prevent severe late‐stage kidney damage (16 weeks) from masking *Gpx3*‐knockout‐induced alterations, mice were sacrificed at 8 weeks post‐modeling to collect serum and renal tissues. *Gpx3* knockout exacerbated renal dysfunction (elevated creatinine/BUN with no significant effect on serum uric acid levels), fibrosis, and tubular VCAM1 expression (Figure 4L–O; Figure S5F), and increased renal ROS (Figure 4P). In summary, these results define a GPX3‐dependent differentiation axis in which GPX3 loss, via oxidative stress, licenses the transition of precursor PTECs into pathogenic VCAM1^+^ Profib PT cells.

### 2.4 Selenium Supplementation Attenuates Fibrosis by Restoring the GPX3‐VCAM1 Axis

**FIGURE 5:**
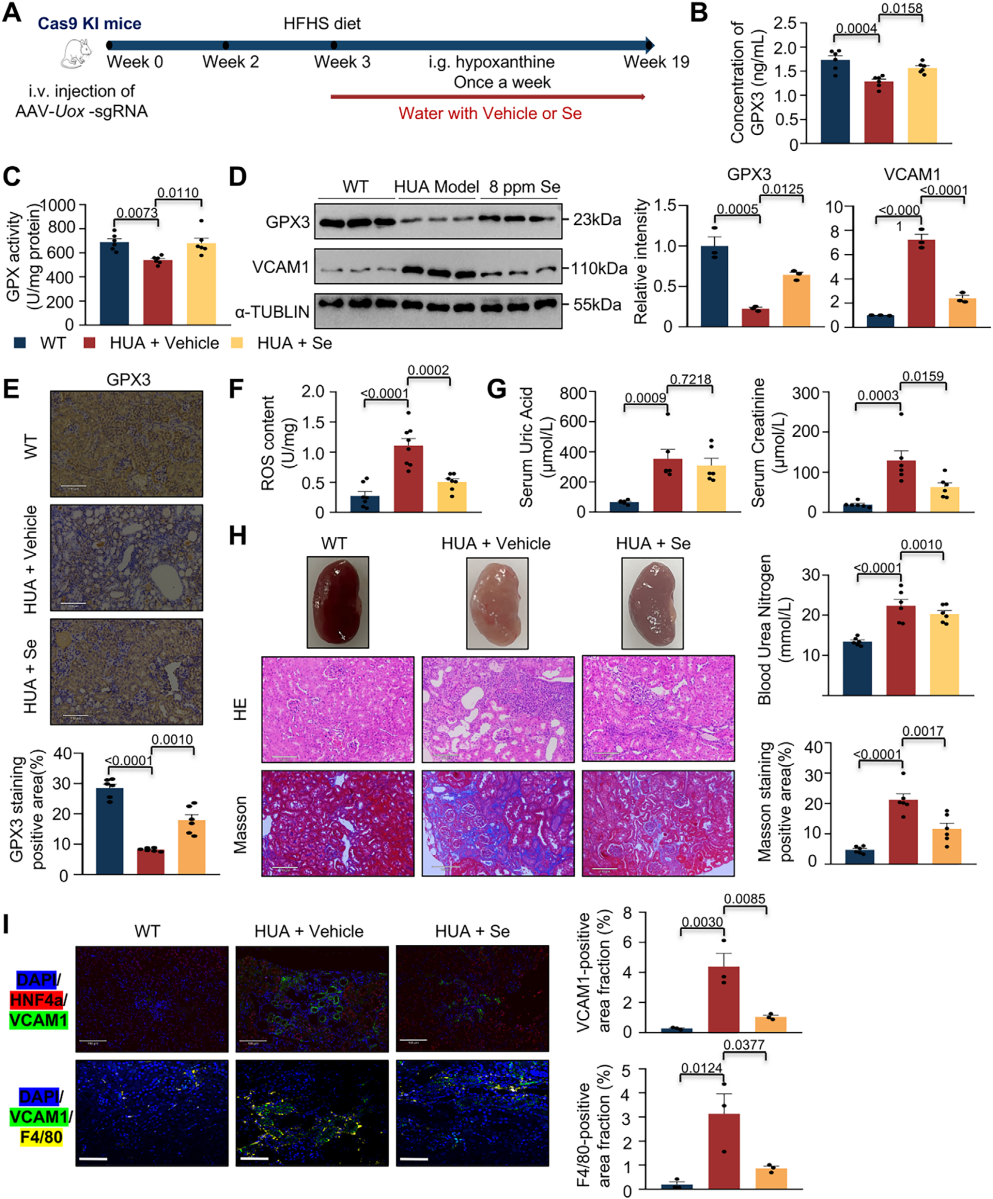
Selenium supplementation rescues GPX3 expression and attenuates hyperuricemic nephropathy. (A) Schematic of the experimental design. Cas9 knock‐in mice were injected intravenously with AAV‐*Uox*‐sgRNA to establish hyperuricemia and fed a high‐fat/high‐sucrose (HFHS) diet. Mice received weekly intragastric administration of hypoxanthine, together with drinking water containing vehicle or selenium (Se) supplementation. (B) Serum GPX3 concentration was measured in wild‐type (WT), hyperuricemia model mice receiving vehicle (HUA + Vehicle), and hyperuricemia model mice receiving selenium supplementation (HUA + Se) (*n* = 6). Statistical differences among groups were analyzed using one‐way ANOVA followed by Tukey's post hoc test (*p* = 0.0005). (C) GPX enzymatic activity in kidney tissues from the indicated groups (*n* = 6). Statistical differences among groups were analyzed using ordinary one‐way ANOVA followed by Tukey's post hoc test (*p* = 0.0042). (D) Western blot analysis and quantification of GPX3 and VCAM1 expression in kidney tissues from WT, HUA model, and HUA mice treated with selenium (8 ppm) (n = 3). Statistical differences among groups were analyzed using ordinary one‐way ANOVA followed by Tukey's post hoc test (*p* = 0.0007 for GPX3 expression; *p* < 0.0001 for VCAM1 expression). (E) Representative immunohistochemical staining and corresponding quantification of GPX3 in kidney sections from the indicated groups (*n* = 6). Scale bar: 110 µm. Statistical differences among groups were analyzed using ordinary one‐way ANOVA followed by Tukey's post hoc test (*p* < 0.0001). (F) Quantification of renal reactive oxygen species (ROS) content in the indicated groups (*n* = 7–8). Statistical differences among groups were analyzed using ordinary one‐way ANOVA followed by Tukey's post hoc test (*p* < 0.0001). (G) Serum biochemical analyses showing serum uric acid, serum creatinine, and blood urea nitrogen (BUN) levels (*n* = 6). Statistical differences among groups were analyzed using ordinary one‐way ANOVA followed by Tukey's post hoc test (*p* = 0.0009 for serum uric acid; *p* = 0.0006 for serum creatinine; *p* < 0.0001 for BUN). (H) Representative gross kidney morphology and histological assessment by hematoxylin and eosin (H&E), and Masson's trichrome staining and corresponding quantification, demonstrating that selenium supplementation alleviates hyperuricemia‐induced renal structural damage and fibrosis (*n* = 6). Scale bar: 110 µm. Statistical differences among groups were analyzed using ordinary one‐way ANOVA followed by Tukey's post hoc test (*p* < 0.0001). (I) Representative immunofluorescence staining and corresponding quantification showing colocalization of VCAM1 with the proximal tubular epithelial marker HNF4A (Scale bar: 100 µm), and VCAM1 with the macrophage marker F4/80 (Scale bar: 50 µm), indicating reduced VCAM1 expression and macrophage infiltration following selenium supplementation (*n* = 3). Statistical differences among groups were analyzed using ordinary one‐way ANOVA followed by Tukey's post hoc test (p = 0.0028 for colocalization of VCAM1 with HNF4a; p = 0.0122 for colocalization of VCAM1 with F4/80).

Given that GPX3 loss drives Profib PT differentiation, we hypothesized that restoring GPX3 activity could be therapeutic. We provided HUA mice with dietary selenium supplementation, a cofactor essential for GPX3 biosynthesis and function (Figure 5A).

Selenium treatment successfully increased circulating GPX3 levels and renal GPX enzymatic activity (Figure 5B,C), and restored GPX3 protein expression in kidney tissue (Figure 5D,E). This intervention concomitantly suppressed the Profib PT phenotype, significantly reducing renal VCAM1 expression (Figure 5D). Selenium also decreased renal reactive oxygen species (ROS) (Figure 5F) and improved renal function, lowering serum creatinine and BUN without substantially affecting serum uric acid (Figure 5G). Histologically, selenium supplementation markedly attenuated renal structural damage, interstitial fibrosis (Figure 5H), VCAM1 expression in proximal tubules, and macrophage infiltration (Figure 5I).

To define the upstream signaling converging on this pathway, we performed bulk RNA‐seq on kidneys from HUA mice treated with selenium, VCAM1 blockade, or MCC950. We identified 151 commonly downregulated genes across all treatments (Figure S6A), enriched for inflammatory and pro‐fibrotic pathways, with NF‐κB signaling as a prominent shared node (Figure S6B). Single‐cell regulon analysis confirmed elevated NFKB1 activity specifically in Profib PT cells (Figure S6C). Furthermore, motif analysis identified a canonical NFKB1 binding site in the *Vcam1* regulatory region (Figure S6D), suggesting NF‐κB may directly regulate *Vcam1* transcription.

In summary, dietary selenium supplementation ameliorates hyperuricemic nephropathy by rescuing GPX3 activity, suppressing oxidative stress and the downstream GPX3‐VCAM1 axis, and attenuating renal inflammation and fibrosis.

## 3 Discussion

This study delineates a novel pathogenic axis in hyperuricemic nephropathy, linking oxidative stress to the emergence of a specific pro‐fibrotic tubular subpopulation and revealing a translatable therapeutic strategy. We first established a robust, multi‐hit mouse model that recapitulates the sustained hyperuricemia, metabolic dysregulation, and progressive renal fibrosis observed in patients. Using this platform, we confirmed the contributory role of NLRP3 inflammasome activation in the kidney. Our central advance, however, lies in the application of single‐nucleus transcriptomics to identify a unique pro‐fibrotic proximal tubule cell (Profib PT) population and to define the GPX3‐VCAM1 axis as its master regulator. Critically, we demonstrate that dietary selenium supplementation, by restoring GPX3, disrupts this axis and confers significant renoprotection.

Our model addresses a critical need in the field. Many previous studies relied on the uricase inhibitor oxonic acid or extreme purine loading, which can cause variable or transient hyperuricemia and introduce extra‐renal toxicities [27]. In contrast, our model—combining liver‐specific *Uox* knockout with a moderate metabolic challenge—generates a stable, genetically defined hyperuricemia that more faithfully recapitulates human pathophysiology, including sex‐specific differences. This provides a superior platform for mechanistic and therapeutic interrogation. The model's additional recapitulation of gut dysbiosis and systemic metabolic reprogramming further enhances its translational relevance, as these features are increasingly implicated in human CKD progression.

While recent single‐cell studies have identified distinct “failed‐repair” or injury‐associated tubular states in models of acute kidney injury (AKI) and its transition to CKD [23, 26], our discovery of the Profib PT cell is significant in several respects. First, it emerges in the context of a pure metabolic insult—chronic hyperuricemia—in the absence of an initial ischemic or obstructive injury, highlighting the proximal tubule's capacity for maladaptive reprogramming under sustained biochemical stress. Second, the conservation of a *VCAM1*‐high PT state in human diabetic kidney disease [35] suggests this may represent a convergent, deleterious response to diverse metabolic injuries, extending the relevance of our findings beyond hyperuricemia. Critically, we move beyond descriptive characterization to establish a precise regulatory mechanism. Our result suggests that the Profib PT state is not merely a static marker of injury but the product of a defined differentiation trajectory from precursor cells, a process gated by the antioxidant enzyme GPX3. Notably, however, this lineage transition is primarily inferred by pseudotime analysis, which captures transcriptional similarity rather than definitive lineage specification. Thus, the precise nature of this cell state transition and its underlying regulatory mechanisms require further experimental validation.

The identification of GPX3 as the key regulator restraining Profib PT differentiation provides a mechanistic link between the well‐documented oxidative stress of hyperuricemia and the execution of a pro‐fibrotic cellular program. Our data show that GPX3 loss, whether through transcriptional downregulation in disease or genetic deletion, creates a permissive redox environment that licenses *VCAM1* upregulation. This positions oxidative stress not as a general bystander of injury but as a specific instructive signal for cell fate decisions within the tubular epithelium. The in vivo exacerbation of fibrosis upon proximal tubule–specific *Gpx3* deletion causally establishes its non‐redundant, cell‐autonomous protective role.

Our study demonstrates a significant decrease in GPX3 expression in PTEC cells following uric acid exposure; however, the precise molecular mechanism by which uric acid downregulates GPX3 remains to be fully elucidated. Although the biological effects of soluble uric acid have often been broadly attributed to its induction of oxidative stress [44, 45], it is increasingly recognized as a specific signaling molecule. This emerging paradigm is supported by recent discoveries identifying direct molecular targets of uric acid, such as UMPS [46] and CD38 [47]. Future studies interrogating whether uric acid directly modulates GPX3 transcription, translation, or stability through such specific receptor‐mediated or enzymatic pathways will be crucial to define this upstream node in the hyperuricemic injury cascade.

The translational power of this mechanistic insight is demonstrated by the efficacy of selenium supplementation. While selenium deficiency is associated with complications in CKD patients—particularly those on hemodialysis [48, 49, 50]—and selenium is a known essential cofactor for GPX3 biosynthesis, evidence for the renal benefits of selenium repletion has remained elusive, especially for hyperuricemic nephropathy. In this study, dietary selenium restoration in the HUA model effectively increased GPX3 activity, suppressed the Profib PT program, reduced macrophage adhesion, and conferred significant renoprotection, independent of serum urate reduction. This finding is crucial, as it suggests that targeting the downstream GPX3‐VCAM1 may be therapeutic even when urate‐lowering therapy is insufficient or poorly tolerated. Furthermore, integrated transcriptomic analysis indicates that suppression of NF‐κB signaling is a common downstream effect of selenium, VCAM1 blockade, and NLRP3 inhibition, revealing a convergent pathway that warrants future mechanistic investigation.

Several limitations should be noted. First, our snRNA‐seq analysis was performed on a limited number of samples (*n* = 1 per condition: WT, HUA, and MCC950). Consequently, the sequencing data were treated as exploratory and hypothesis‐generating rather than as statistically powered comparisons, and key findings were subsequently validated using orthogonal approaches with independent biological replicates. Second, while the Profib PT signature is conserved in human diabetic kidney disease, its specific presence and role in human hyperuricemic nephropathy require direct validation in patient biopsies. Third, the precise molecular circuitry linking GPX3 loss, ROS, and NF‐κB activation to *Vcam1* transcription warrants further elucidation. Finally, the long‐term efficacy and safety of selenium supplementation for renal protection in chronic settings need to be evaluated in clinical studies.

**FIGURE 6:**
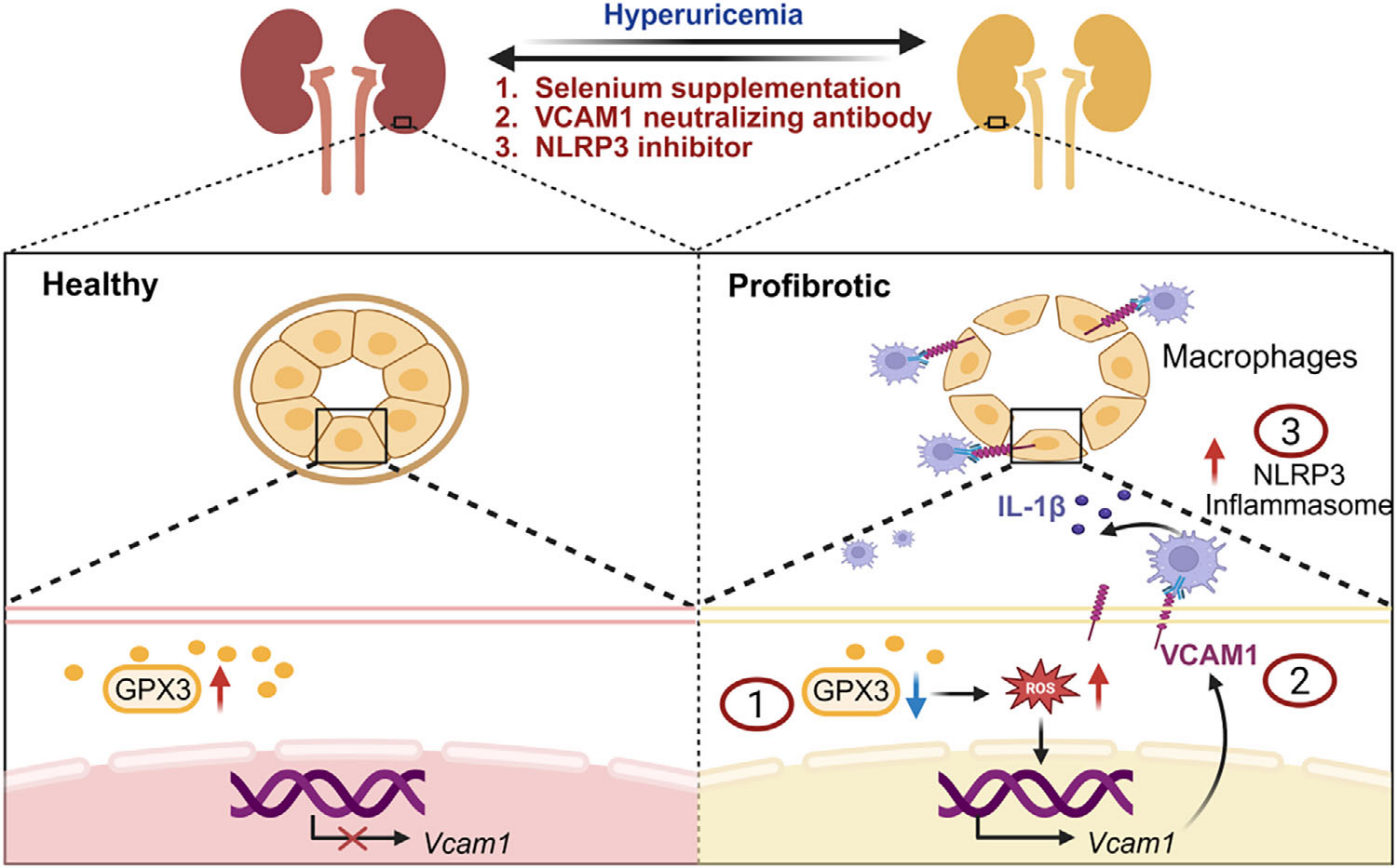
Schematic model summarizing the proposed mechanism. Hyperuricemia induces NLRP3 inflammasome signaling activation and downregulation of GPX3 in proximal tubular epithelial cells, leading to oxidative stress‐driven VCAM1 upregulation, emergence of pro‐fibrotic proximal tubule cells, macrophage recruitment, and renal fibrosis. Therapeutic interventions, including selenium supplementation, VCAM1 neutralization, and NLRP3 inhibition, mitigate renal inflammation and fibrosis by restoring GPX3 expression or interrupting downstream inflammatory signaling. The graphical summary cartoon was created with BioRender (https://app.biorender.com).

In conclusion, our study moves the field beyond the established paradigm of urate crystal and NLRP3‐mediated inflammation. We unveil a novel pathologic axis centered on GPX3 deficiency in proximal tubules, which licenses the differentiation of a pro‐fibrotic, VCAM1‐expressing cell state that drives inflammation and fibrosis (Figure 6). These findings position the GPX3‐VCAM1 axis as a promising new therapeutic target. Dietary selenium supplementation, a simple and low‐risk nutritional intervention, emerges as a compelling strategy to rectify this axis and mitigate kidney injury, offering a novel adjunctive approach to the management of hyperuricemic nephropathy.

## 4 Materials and Methods

### 4.1 Animals

Animal studies were approved by the Institutional Animal Care and Use Committee of the Shanghai Institute for Nutrition and Health (ethical committee approval no. SINH‐2024‐DQR‐3). All animals were healthy, maintained on a C57BL/6 background, and aged 8–12 weeks at the time of experimentation. Mice were housed under specific pathogen‐free (SPF) conditions with a 12‐h light/dark cycle at 22–25°C and 40–60% relative humidity and had ad libitum access to food and water.

The colony of Cre‐dependent Cas9 knockin mice (B6;129­Gt(ROSA)26Sor ^1(CAG­cas9*,­EGFP) Fezh^/J, The Jackson Laboratory, Stock No. 024857) was maintained by crossing with wild‐type C57BL/6J mice (Shanghai Laboratory Animal Center).

To establish the hyperuricemia mouse model, liver‐specific knockout of urate oxidase (Uox) was achieved in Cas9 knock‐in mice via tail‐vein intravenous injection of AAV8‐TBG‐Cre‐Uox‐sgRNA as previously described [51]. Mice were orally administered hypoxanthine (300 mg/kg, MCE, HY‐N0091) once weekly for 16 weeks, starting three weeks after AAV‐mediated hepatic *Uox* depletion. Control mice received 0.5% CMC‐Na (MCE, HY‐Y1889A) as vehicle treatment. Mice were fed a high‐fat/high‐sucrose (HFHS) diet (Research Diets, D12327) beginning at the time of AAV injection and maintained throughout the entire experimental period.

To investigate the role of NLRP3 inflammasome signaling activation on hyperuricemic nephropathy, mice received intraperitoneal injections of MCC950 (20 mg/kg) (MCE, HY‐12815) starting three weeks after AAV‐mediated hepatic *Uox* depletion and administered twice per week. Animals administered an equal volume of vehicle solution (phosphate‐buffered saline, PBS) were used in control groups.

For VCAM1 blockade, HUA mice were randomly assigned to either the HUA‐IgG group or the HUA‐VCAM1ab group at eight weeks after hypoxanthine administration. Mice in the HUA‐VCAM1ab group received intraperitoneal injections of a VCAM1‐neutralizing antibody (BioXCell, BE0027) at a dose of 10 mg/kg twice per week for eight weeks, whereas WT and HUA‐IgG mice were administered an equivalent dose of isotype control IgG (BioXCell, BE0088).

For the hyperuricemia model with renal tubular epithelial cell‐specific *Gpx3* knockout mice, adeno‐associated virus 9 (AAV9) vectors expressing Cre recombinase and *Gpx3*‐sgRNA (5’‐GTCAACGTAGCCAGCTACTG‐3’+AGG) were provided by Hanheng Biotechnology (Shanghai, China). Briefly, Cas9 knock‐in mice were injected with 1 × 10^12^ vector genomes (vg)/mL AAV9‐Ksp‐Cre‐*Gpx3*‐sgRNA and 1 × 10^12^ vg/ml AAV9‐Ksp‐Cre‐Ctrl into 5 different sites (10 µL at each site) of the renal cortex with a glass micropipette after AAV8‐TBG‐Cre‐*Uox*‐sgRNA delivery by tail vein injection. To avoid severe and irreversible renal damage at the late stage (16 weeks) that might mask the phenotypic and functional alterations induced by *Gpx3* knockout, mice were sacrificed at an optimal intermediate time point of 8 weeks post‐modeling to harvest serum and renal tissues for further analysis.

For selenium supplementation, mice were provided drinking water containing sodium selenite (Na_2_SeO_3_) (Sigma, 214485) at a selenium concentration of 8 ppm, while WT and HUA Model mice received drinking water supplemented with the corresponding vehicle. Selenium‐ and vehicle‐containing water was supplied continuously after hypoxanthine administration.

### 4.2 Screening of *Uox* sgRNAs and Validation of *Uox* Knockout Efficiency

Candidate single‐guide RNAs (sgRNAs) targeting the mouse *Uox* gene were first evaluated in cultured NIH3T3 cells using a T7 endonuclease I (T7E1) mismatch cleavage assay. Briefly, cells were transfected with constructs expressing Cas9 together with the indicated *Uox* sgRNAs. Genomic DNA was subsequently extracted, and the region surrounding the corresponding sgRNA target site was amplified by PCR. The PCR products were denatured and reannealed to generate heteroduplexes, followed by digestion with T7E1. The cleavage products were separated by agarose gel electrophoresis, and genome‐editing activity was estimated based on the intensity of the cleaved DNA fragments. Among the three candidate sgRNAs, sgRNA1 exhibited the highest editing efficiency and was therefore selected for subsequent in vivo experiments. The target sequence of *Uox* sgRNA1 was 5′‐GTTCTCCATATTCAGAGAGA‐3′, targeting exon 2 of the *Uox* gene.

To validate the efficiency of *Uox* depletion in vivo, liver tissues were collected from wild‐type and hyperuricemia model mice. Total RNA was isolated from liver tissues, and *Uox* mRNA expression was measured by quantitative reverse‐transcription PCR. In parallel, liver proteins were extracted and subjected to Western blotting to determine UOX protein abundance, with GAPDH used as the loading control.

### 4.3 Kidney Tissue Collection and Nuclear Dissociation

Mice were euthanized by intraperitoneal injection of 4% chloral hydrate in accordance with institutional guidelines. Kidneys were rapidly harvested and immediately frozen at ‐80°C for storage. The renal cortex was carefully dissected from the frozen samples and cut into small pieces (<0.5 cm), and immediately transferred into a glass Dounce tissue grinder (Sigma, D8938). The tissue was homogenized in 2 mL of ice‐cold Nuclei EZ lysis buffer by 25 strokes with pestle A followed by 25 strokes with pestle B. The homogenate was incubated on ice for 5 min, after which an additional 3 mL of cold EZ lysis buffer was added, and the sample was gently mixed. Nuclei were pelleted by centrifugation at 500 × *g* for 5 min at 4°C, resuspended in 5 mL ice‐cold EZ lysis buffer, and incubated on ice for another 5 min. After centrifugation, the nuclear pellet was washed with 5 mL nuclei suspension buffer (NSB; composed of 1× PBS, 0.01% BSA, and 0.1% RNase inhibitor (Clontech, 2313A)). Isolated nuclei were resuspended in 2 mL NSB, filtered through a 35 µm cell strainer (Corning‐Falcon, 352235), and counted using a hemocytometer. The final nuclear concentration was adjusted to 1,000 nuclei per µL and loaded on a 10× channel.

### 4.4 10x Library Preparation and Sequencing

Single‐nucleus RNA sequencing libraries were prepared using the Chromium Next GEM Single Cell 3′ Library Kit v3.1 (10x Genomics, 1000157) following the manufacturer's protocol. Nuclei were loaded onto the Chromium Controller to generate Gel Beads‐in‐Emulsion (GEMs), where polyadenylated RNA was reverse‐transcribed and barcoded with cell‐specific barcodes and unique molecular identifiers (UMIs). Barcoded cDNA was amplified, and sequencing libraries were constructed according to standard 10x Genomics procedures. Sample indexing was performed using the Chromium Next GEM Single Cell 3′ GEM Kit v3.1 (10x Genomics, 1000123). Libraries were quantified using an Agilent Bioanalyzer 2100 and Qubit High Sensitivity DNA Assay and sequenced on an Illumina NovaSeq 6000 platform using paired‐end 2 × 150 bp chemistry. Library preparation and sequencing were performed by Shanghai Majorbio Bio‐pharm Technology Co., Ltd.

### 4.5 Single Nucleus RNA‐seq Data Processing

Single‐nucleus RNA‐seq data from three samples (WT, HUA model, and MCC950) were processed with Cell Ranger v7.1.0 (10x Genomics) using default parameters. Illumina FASTQ reads were aligned to the mouse genome (GRCm39) with STAR, and UMI‐based gene‐barcode matrices were generated after filtering non‐cell‐associated barcodes. In total, 307.73 Gb of raw data were obtained, yielding 44,555 nuclei (13,838 WT; 14,521 HUA model; 16,196 MCC950). The matrices were imported into Seurat (v4.1.1) for downstream analysis.

Quality control was then performed. Low‐quality nuclei were removed based on UMI counts, detected genes, and mitochondrial content (outliers beyond mean ± 2 SD), with mitochondrial percentages computed by PercentageFeatureSet; ribosomal content, erythrocyte‐associated signals, and doublets were also assessed and filtered. After QC, 11,469 (WT), 12,081 (HUA model), and 12,960 (MCC950) nuclei were retained.

### 4.6 Dimensionality Reduction, Clustering, and Cell‐Type Annotation (UMAP)

Cells were integrated with Seurat (FindIntegrationAnchors/IntegrateData), scaled, and analyzed by PCA. Clustering was performed on an SNN graph built from the top 30 principal components using the Louvain algorithm, visualized by UMAP, and cell types were annotated using Seurat marker genes (Wilcoxon test) and SCINA with canonical markers.

### 4.7 Differential Expression Analysis and KEGG Pathway Enrichment

Differentially expressed genes (DEGs) between groups (samples or clusters) were identified in Seurat using the FindMarkers function with a likelihood ratio test. Genes with |log2FC| > 0.25 and an adjusted *p*‐value ≤ 0.05 were considered significant. KEGG pathway enrichment analysis was then performed on significant DEGs using Python‐based workflows, and pathways with Bonferroni‐corrected *p*‐value ≤ 0.05 were considered significantly enriched, using all detected genes as the background.

### 4.8 Pseudo‐Time Trajectory Analysis

Pseudo‐time trajectory analysis was performed on proximal tubule (PT) cells using Monocle2 (v.2.22.0). Genes expressed in ≥10 cells, with a mean expression ≥0.1 and dispersion ≥2, were selected. Differential expression analysis (FDR < 0.05) was used to identify trajectory‐defining genes. Following dimensionality reduction and minimum spanning tree construction, cells were ordered along a pseudotime trajectory, and smoothing was applied via smoothData(). Genes significantly associated with pseudotime (*p* < 0.01) were identified using differentialGeneTest, and branch‐dependent genes were analyzed with the BEAM function.

### 4.9 SCENIC Analysis for Transcription Factor Regulon Inference

Transcription factor (TF) regulatory network activity was inferred using the SCENIC workflow. UMI counts from transcriptionally defined fibroblasts were used as input. We constructed and filtered TF‐target co‐expression modules by target gene importance. Genes with significant motif enrichment were designated as direct TF targets and defined as regulons (using default parameters). Regulon activity per cell was quantified with AUCell, generating a regulon activity matrix. This matrix was used for cell re‐clustering, with regulons classified as “active” based on default thresholds. The matrix was also imported into Seurat for visualization and comparative analysis. Data analysis was conducted on the Majorbio Cloud Platform (www.majorbio.com).

### 4.10 Integration of Present Dataset With Publicly Available Data

To understand similarities and differences with the publicly available human early diabetic kidney disease (DKD) dataset (GSE131882), we performed an integrated analysis. Both datasets (present and published) were realigned using Cell Ranger v7.1.0 (10x Genomics), and then the filtered matrix output was used to make a Seurat object using Seurat (v.5.0.1). Filtering, data normalization, and unbiased clustering were performed on the merged dataset as described above.

### 4.11 Mouse Bulk RNA‐seq Analysis

Total RNA was extracted using TRIzol reagent (Thermo Fisher Scientific, 15596018CN) according to the manufacturer's instructions. Sequencing libraries were constructed using the Illumina Stranded mRNA Preparation, Ligation (San Diego, CA). The sequencing library was sequenced on the NovaSeq X Plus platform (PE150) using the NovaSeq Reagent Kit. Three independent biological replicates were included in each group. Reads were aligned to the GENCODE mouse reference genome (GRCm39) using STAR (v2.7.1a). TPM values were calculated to represent relative gene expression levels, whereas raw read counts were used for differential expression analysis. Differentially expressed genes (DEGs) were identified using DESeq2, in which raw read counts were internally normalized using the median‐of‐ratios method based on size factors. The statistical design was specified as ∼ group, with no additional batch covariates included because all samples were processed and sequenced in a single batch without known confounding batch effects. Genes with an absolute log2 fold change (|log2FC|) ≥ 1 and a Benjamini–Hochberg‐adjusted *p*‐value (FDR) ≤ 0.05 were considered significantly differentially expressed. KEGG pathway enrichment analysis of DEGs was performed using KOBAS against the whole‐transcriptome background, and pathways with a Bonferroni‐corrected *p*‐value ≤ 0.05 were considered significantly enriched. Data analysis was performed using the Majorbio Cloud Platform (www.majorbio.com).

### 4.12 Serum Untargeted Metabolomics

Blood samples harvested from the tail vein were centrifuged at 14,000 rpm for 10 min at 4°C to isolate serum, which was subsequently cryopreserved at −80°C. For metabolite extraction, 3 µL of thawed serum was homogenized with 120 µL of pure methanol. Following thorough vortexing, the mixture underwent two consecutive centrifugation rounds (14,000 rpm, 4°C, 30 min each). The resulting supernatant was retrieved for downstream LC‐MS analysis. LC‐MS methods were performed using a Vanquish Ultra‐Performance Liquid Chromatography (UPLC) system coupled to a Q‐Exactive Plus Orbitrap mass spectrometer (Thermo Fisher Scientific). Separation was achieved on a HILIC column (2.1 mm × 150 mm, 5 µm; HILICON) maintained at 25°C. The mobile phase system comprised solvent A (water:acetonitrile 95:5, containing 20 mM ammonium acetate and 20 mM ammonium hydroxide, pH 9.4) and solvent B (100% acetonitrile). The elution gradient was programmed as follows: 0–2 min, 95% B; 3–7 min, 75% B; 8–9 min, 70% B; 10–12 min, 50% B; 13–14 min, 25% B; 16–20.5 min, 0% B; and 21–28 min, 90% B. A 5 µL sample volume was injected for analysis. Mass spectrometry data were acquired in polarity‐switching mode (positive/negative) with a scan range of m/z 60–900. Key MS settings included a resolution of 140,000 (at m/z 200), an AGC target of 3 × 10^6^, and a maximum injection time of 200 ms. Data processing was conducted using El‐Maven software.

### 4.13 Mouse Fecal 16S rRNA Gene Sequencing

Fresh mouse fecal pellets were collected under sterile conditions, snap‐frozen, and stored at −80°C. Microbial DNA was extracted using the E.Z.N.A. soil DNA Kit (Omega Bio‐Tek, Norcross, GA, U.S.) according to the manufacturer's instructions. The bacterial 16S rRNA gene hypervariable region was amplified by PCR using universal primers, and purified amplicons were used to construct indexed libraries. Libraries were sequenced on an Illumina Nextseq2000 platform (Illumina, San Diego, USA) according to the standard protocols by Majorbio Bio‐Pharm Technology Co. Ltd. (Shanghai, China) with paired‐end reads. Data analysis was performed using the Majorbio Cloud Platform (www.majorbio.com).

### 4.14 Cell Culture and Treatments

HK‐2 cells (Cell Bank, Type Culture Collection Committee, Chinese Academy of Sciences) were cultured in Keratinocyte‐SFM (Gibco, 17005042) supplemented with epidermal growth factor (EGF) and bovine pituitary extract (BPE) (according to the manufacturer's instructions) and 1% penicillin‐streptomycin (YEASEN, 60162ES76). Cells were maintained at 37°C in a humidified incubator with 5% CO_2_.

For CRISPR‐Cas9‐mediated *GPX3* knockout in HK2 cells, single guide RNAs (sgRNAs) targeting human *GPX3* (AGATGTACCCAGGAGCTCCG) and a non‐targeting control sgRNA were produced by MiaoLingBio (Shanghai, China). Lentiviral vectors encoding Cas9 and the sgRNA were packaged to produce viral particles and used to transduce HK2 cells. After 24 h, the transduced cells were selected using 2 ug/mL puromycin for 7 days to establish stable cell lines. Genome editing efficiency was validated by Western blotting before downstream experiments.

For uric acid (UA) treatment, a 1200 µm UA working solution was prepared by weighing uric acid powder (MCE, HY‐B2130) and dissolving it in culture medium containing 10% bovine serum albumin (BSA) with ultrasonic sonication until fully solubilized. HK2 cells were cultured in UA‐containing medium (1200 µm) for 5 days, with the medium replaced once during the treatment period. Control cells were cultured in the same medium containing 10% BSA without UA.

To investigate whether GPX3 restrains VCAM1 through redox regulation, HK2 cells were administered with the antioxidant N‐acetylcysteine (NAC) (Selleck, S1623) in the presence or absence of UA or *Gpx3* sgRNA for 5 days.

### 4.15 Serum Uric Acid, Creatinine and Blood Urea Nitrogen Measurements

Blood samples were collected, allowed to clot at room temperature, and then centrifuged at 4°C (6,000 × *g*, 10 min) to obtain serum. Uric acid was determined using a uric acid detection kit (Nanjing Jiancheng Bioengineering Institute, C012‐2‐1). Serum creatinine was determined using a creatinine detection kit (Nanjing Jiancheng Bioengineering Institute, C011‐2‐1). BUN was determined using a BUN detection kit (Nanjing Jiancheng Bioengineering Institute, C013‐2‐1). For the hyperuricemia model with renal tubular epithelial cell‐specific *Gpx3* knockout mice, serum uric acid, creatinine, and blood urea nitrogen were quantified using a clinical chemistry analyzer DRI‐CHEM NX700V (FUJIFILM).

### 4.16 GPX Activity Assay in Mouse Kidney Tissue

Glutathione peroxidase (GPX/GSH‐Px) activity in mouse kidney tissues was measured using the Glutathione Peroxidase (GSH‐PX) Assay Kit (colorimetric method) (Nanjing Jiancheng Bioengineering Institute, A005‐1‐2) according to the manufacturer's instructions. Briefly, kidney tissues were homogenized in ice‐cold lysis buffer, and the supernatants were collected by centrifugation. GPX activity was determined colorimetrically, and results were normalized to total protein concentration measured by a BCA assay (ThermoFisher, 23225).

### 4.17 ELISA Measurement of GPX3 and ROS Levels in Mouse Kidney Tissue

Mouse kidney tissues were harvested and homogenized in ice‐cold PBS (or the sample diluent provided with the kits). Homogenates were centrifuged to remove debris, and the supernatants were collected for ELISA assays. Renal GPX3 levels were measured using a Glutathione Peroxidase 3 (GPX3) ELISA kit (Nanjing Jiancheng Bioengineering Institute, H576) according to the manufacturer's instructions. Renal ROS levels were determined using a Mouse Reactive Oxygen Species (ROS) ELISA Kit (ABclonal, RK04770) following the manufacturer's protocol. Absorbance was read on a microplate reader, and concentrations were calculated from standard curves. Where indicated, results were normalized to total protein concentration measured by a BCA assay (ThermoFisher, 23225).

### 4.18 Intracellular ROS Measurement by Flow Cytometry

Intracellular reactive oxygen species (ROS) levels were measured using a Reactive Oxygen Species (ROS) Assay Kit (Servicebio, G1706) according to the manufacturer's instructions. Briefly, cells were collected, washed with PBS, and incubated with the ROS fluorescent probe working solution at 37°C in the dark for 30 min. After incubation, cells were washed to remove excess dye, resuspended in PBS, and immediately analyzed by flow cytometry. ROS levels were quantified as the percentage of ROS‐positive cells, and data were analyzed using FlowJo.

### 4.19 Histology and Immunohistochemistry

Mouse kidney tissues were fixed in 4% paraformaldehyde and embedded in paraffin following standard procedures for haematoxylin and eosin(H&E) and Masson's trichrome staining (Servicebio). For detection of α‐SMA (1:500, Servicebio, GB111364), NLRP3 (1:300, Servicebio, GB114320), F4/80 (1:500, Cell Signaling Technology, 70076S) and GPX3 (1:500, ABCAM, ab256470), immunostaining was performed via standard protocols (Servicebio). Quantitative analysis of the histological and immunohistochemical staining images was performed using ImageJ software.

### 4.20 Immunofluorescence Staining

Formalin‐fixed paraffin‐embedded (FFPE) tissue sections were deparaffinized and subjected to antigen retrieval, then blocked with 1% bovine serum albumin (BSA) and permeabilized with 0.1% Triton X‐100 in PBS. Sections were incubated overnight at 4°C with primary antibodies against VCAM1 (1:1000, Servicebio, GB113498), HNF4A (1:500, Servicebio, GB115549), and F4/80 (1:500, Cell Signaling Technology, 70076S), followed by incubation with species‐matched fluorescent secondary antibodies at the appropriate dilution. Nuclei were counterstained with DAPI, and slides were mounted in Prolong Gold (Life Technologies). Immunostaining was performed via standard protocols (Servicebio). Images were acquired using an Olympus confocal microscope (high‐content cell imaging analyzer). Quantitative analysis of the immunofluorescence staining images was performed using ImageJ software.

### 4.21 Western Blotting

Protein from cells or tissues was extracted using RIPA buffer (Millipore, 20188) and was subjected to Western blotting. The primary antibodies used in the experiments were antibodies to UOX (Santa Cruz Biotechnology, sc‐166214), GPX3 (ABCAM, ab256470), VCAM1 (Servicebio, GB113498), and α‐TUBLIN (Proteintech, 11224‐1‐AP). Densitometric quantification of the protein bands was performed using ImageJ software.

### 4.22 Data Statistics and Analysis

The data were expressed as mean ± s.e.m. Statistical differences between different groups were analyzed using the unpaired two‐tailed *t*‐test, ordinary one‐way ANOVA, or Two‐way ANOVA as indicated in the figure legends. Statistical significance was defined as *p* < 0.05. Exact *p*‐values are displayed in the corresponding figures, and the overall ANOVA *p*‐values have been added to the relevant figure legends. *p*‐Values were calculated in GraphPad Prism 8.0. All sequencing data analyses were performed using the Majorbio Cloud Platform (www.majorbio.com).

## Author Contributions

Q.D. and Y.Q. conceived and designed the project; Y.Q, Q.Z., Y.L., Y.Y., T.Z., Y.Y., D.W., Y.T., and Y.L. performed the experiments and analyzed data; Y.Q. and Q.D. wrote the manuscript; Q.D. and C.J. supervised the project.

## Funding

This work was supported by grants from the Program of EnShi Tujia&Miao Autonomous Prefecture Bureau of Scientific & Technological Affairs, the Noncommunicable Chronic Diseases‐National Science and Technology Major Project (2023ZD0507700), the National Key R&D Program of China (2023YFA1801100), the National Natural Science Foundation of China (92357306, 82330028, 82425013), the Strategic Priority Research Program of the Chinese Academy of Sciences (XDB1150201), the Key R&D Program of Shandong Province, China (2025CXPT176), and the Research Project of Jinan Microecological Biomedicine Shandong Laboratory (2025008B, 2025009B, 2025010B, 2025011B).

## Ethics Statement

Animal studies were approved by the Institutional Animal Care and Use Committee of the Shanghai Institute for Nutrition and Health (ethical committee approval no. SINH‐2024‐DQR‐3).

## Translational Statement

Hyperuricemia (elevated uric acid) is a widespread metabolic condition that damages kidneys, but treatments beyond lowering uric acid are lacking. We identify a specific type of diseased kidney cell that drives this damage and discover that its formation is controlled by an antioxidant enzyme, GPX3. Crucially, we show that boosting this enzyme with a simple dietary supplement (selenium) blocks the harmful cells and prevents kidney scarring in a preclinical model. This work reveals a new targetable pathway for a common form of kidney disease and suggests a safe, nutritional approach for prevention.

## Conflicts of Interest

The authors declare no conflicts of interest.

## Supporting information




**Supporting File**: advs77676‐sup‐0001‐SuppMat.docx.

## Data Availability

All data are available in the main text or the supplementary materials. All biological materials used are readily available from the authors or from standard commercial sources. The source data required to reanalyze the data included in this report are available from the corresponding author upon request. All sequencing data have been deposited in NODE (http://www.biosino.org/node): RNA‐seq, snRNA‐seq of animal kidneys, 16S rDNA‐seq (OEP00006872).
