# Supplementary material for: The GPX3‐VCAM1 Axis Gates Pro‐Fibrotic Tubule Cell Fate in Hyperuricemic Nephropathy
Source: Adv Sci (Weinh). 2026 Sep 9:e77676. Online ahead of print. doi: 10.1002/advs.77676 (PMC13558898; doi:10.1002/advs.77676)
Supplement: Supplementary file 1 [file ADVS-9999-e77676-s001.docx]

**Figure S1. Neither *Uox* gene disruption nor diet-induced hypoxanthine loading is sufficient to generate a stable hyperuricemia mouse model.**

(A) T7E1 survey assay for screening candidate sgRNAs targeting *Uox*. NIH3T3 cells were transduced with lentiviruses expressing Cas9 and the indicated *Uox* sgRNAs, and genome-editing activity was assessed by T7 endonuclease I cleavage of PCR-amplified target regions. sgRNA1 exhibited the highest genome-editing activity and was selected for subsequent experiments.(B) Schematic diagram illustrating the targeting locus of *Uox* sgRNA, which is designed to bind the sequence in exon 2 (ex2) of the mouse *Uox* gene. The full sgRNA sequence is provided at the top.

(C) Relative hepatic *Uox* mRNA expression in WT and HUA model mice, as determined by quantitative real-time PCR. *Uox* mRNA levels were significantly reduced in the livers of HUA model mice compared with WT mice.

(D) Western blot analysis and quantitative analysis of UOX protein expression in liver tissues from wild-type (WT) mice and established hyperuricemia (HUA) model mice, with GAPDH used as the internal loading control (n = 3). Statistical significance was determined by an unpaired two-tailed t test.

(E) Representative polarized light microscopy images assessing urate crystal deposition. No urate crystals were detected within the renal tubular lumens in HUA model. Scale bar: 100 μm.

(F) Serum biochemical parameters in three groups of mice: wild-type (WT), *Uox*-sgRNA, and WT mice subjected to a high-fat/high-sucrose diet combined with hypoxanthine administration (WT+HFHS+HPX). Shown are serum uric acid, serum creatinine, and blood urea nitrogen (BUN) (n=6). Statistical differences among groups were analyzed using ordinary one-way ANOVA followed by Tukey's post hoc test (P = 0.0007 for serum uric acid; P = 0.2794 for serum creatinine; P = 0.1796 for BUN).

(G) Representative gross morphology of kidneys and histological analysis by hematoxylin and eosin (H&E), and Masson’s trichrome staining and quantification analysis, showing no overt structural or fibrotic alterations across groups (n = 3). Scale bar: 110 μm. Statistical differences among groups were analyzed using ordinary one-way ANOVA followed by Tukey's post hoc test (P = 0.9448).

**Figure S2. Female mice exhibit an attenuated hyperuricemic response compared to males.**

(A) Serum biochemical analyses in female wild-type (Female-WT) and female hyperuricemia model (Female-HUA Model) mice generated using the same protocol as applied in male mice. Serum uric acid levels were significantly elevated in female mice, albeit remaining far below those observed in male hyperuricemic mice, while serum creatinine and blood urea nitrogen (BUN) levels showed no significant differences between groups (n = 9). Statistical significance was determined by an unpaired two-tailed t test.

(B) Representative gross kidney morphology and renal histopathological analysis by hematoxylin and eosin (H&E), and Masson’s trichrome staining and quantification analysis in female mice. No obvious structural abnormalities or fibrotic changes were observed between Female-WT and Female-HUA Model groups (n = 3). Scale bar: 110 μm. Statistical significance was determined by an unpaired two-tailed t test.

**Figure S3.** **Single-nucleus RNA sequencing (snRNA-seq) reveals cellular composition changes and pro-fibrotic proximal tubule subpopulations in hyperuricemic nephropathy.**

(A) UMAP visualization of all renal cell populations identified by single-nucleus RNA sequencing, including endothelial cells (Endo), proximal tubule epithelial cells (PT), thick ascending limb cells (ALOH), mesangial cells, macrophages, distal convoluted tubule cells (DCT), connecting tubule cells (CNT), collecting duct cells (CD), pro-fibrotic proximal tubule cells (Profib PT), distal loop of Henle cells (DLOH), T cells, intercalated cells (IC), dendritic cells (DC), B cells, epithelial cells, and podocytes (Podo).

(B) Violin plots showing expression of representative marker genes across major renal cell types, confirming cell-type annotation accuracy.

(C) Relative proportions of major renal cell populations in wild-type (WT), hyperuricemia (HUA) model, and HUA mice treated with the NLRP3 inhibitor MCC950.

(D) Subclustering and UMAP visualization of proximal tubule epithelial cells from mouse kidneys, identifying Profib PT cells as a distinct injury-associated PT subpopulation.

(E) Heatmap of canonical extracellular matrix and profibrotic marker genes (*Fn1*, *Col4a1*, *Col4a2*, *Spp1*, *Pdgfd*, *Itgb6*, *Itgb8*) across PT subclusters, showing robust upregulation of fibrotic transcripts specifically in Profib PT cells. Color gradient represents normalized gene expression values ranging from 0 (blue) to 1 (red).

(F) Quantitative analysis of VCAM1-positive areas (%) in renal sections from WT and HUA model mice (n = 3). Statistical significance was determined by an unpaired two-tailed t test.

(G) Representative dual immunofluorescence staining and quantitative analysis of WT and HUA renal sections, showing co-localization of LRP2 (proximal tubule marker) with VCAM1, and Collagen I (fibrotic matrix marker) with VCAM1, respectively (n = 6). Scale bar = 10 μm. Statistical significance was determined by an unpaired two-tailed t test.

**Figure S4. Single-nucleus RNA sequencing (snRNA-seq) reveals cellular composition changes and pro-fibrotic proximal tubule subpopulations in hyperuricemic nephropathy.**

(A) UMAP visualization of proximal tubule epithelial cell subpopulations from human early-stage diabetic kidney disease samples, revealing the presence of a Profib PT–like population.

(B) KEGG pathway enrichment analysis of genes upregulated in human Profib PT cells compared with other proximal tubule subpopulations, highlighting pathways related to cell adhesion, extracellular matrix interactions, inflammatory signaling, and cellular senescence.

(C) KEGG pathway enrichment analysis of genes downregulated following VCAM1-neutralizing antibody treatment compared with the HUA model, indicating suppression of pro-fibrotic and pro-inflammatory signaling pathways.

**Figure S5. Pseudotime-dependent decline of GPX3 expression during proximal tubular epithelial cell transcriptional progression and its redox-dependent regulation of VCAM1.**

(A) Heatmap of pseudotime-dependent gene expression in mouse proximal tubular epithelial cells, showing *Gpx3* downregulation along the transcriptional continuum toward injury-associated states.
(B) Heatmap of pseudotime-dependent gene expression in proximal tubular epithelial cells from human early-stage diabetic kidney disease datasets, revealing a similar downregulation pattern of *GPX3* along the inferred transcriptional continuum.

(C) Western blot quantification analysis of GPX3 expression in proximal tubular epithelial cells treated with 1200 μM uric acid (UA), showing UA-induced downregulation of GPX3 (n = 3). Statistical significance was determined by an unpaired two-tailed t test.

(D) Western blot quantification analysis of GPX3 and VCAM1 following *Gpx3* knockout in proximal tubular epithelial cells, demonstrating increased VCAM1 expression upon GPX3 loss (n = 3). Statistical significance was determined by an unpaired two-tailed t test.

(E) Western blot quantification analysis of VCAM1 expression in control, *Gpx3*-knockdown, and *Gpx3*-knockdown + 10 mM NAC groups, demonstrating antioxidant treatment with N-acetylcysteine (NAC) attenuates VCAM1 upregulation induced by Gpx3 knockout (n = 3). Statistical differences among groups were analyzed using ordinary one-way ANOVA followed by Tukey's post hoc test (P < 0.001).

(F) Western blot analysis and quantification analysis of VCAM1 expression in proximal tubular epithelial cells treated with uric acid (UA) in the presence or absence of the antioxidant N-acetylcysteine (NAC), showing that NAC attenuates UA-induced VCAM1 upregulation (n = 3). Statistical differences among groups were analyzed using ordinary one-way ANOVA followed by Tukey's post hoc test (P < 0.001).

(G) Western blot quantification analysis of GPX3 and VCAM1 expression in kidney tissues from control and proximal tubule–specific *Gpx3* knockout mice (n = 4). Statistical significance was determined by an unpaired two-tailed t test.

**Figure S6. NF-κB signaling is potentially involved in regulating GPX3-associated differentiation of VCAM1⁺ pro-fibrotic** **proximal tubular epithelial cells.**

(A) Venn diagram showing downregulated genes identified by bulk RNA sequencing in hyperuricemia (HUA) mice treated with selenium supplementation (Se), VCAM1-neutralizing antibody (VCAM1 ab), or the NLRP3 inhibitor MCC950, each compared with the HUA model group. A total of 151 genes were commonly downregulated across all three interventions.

(B) KEGG pathway enrichment analysis of the 151 shared downregulated genes, highlighting enrichment of inflammatory and pro-fibrotic pathways, with particular emphasis on the NF-κB signaling pathway.

(C) Heatmap showing regulon activity of selected transcription factors across proximal tubular epithelial cells (PTEC) subpopulations based on single-cell regulatory network analysis. Increased NFKB1 regulon activity is observed in Profib PT cells compared with healthy PT subsets, whereas other PT states display lower activity.

(D) Sequence logo representing the NFKB1 binding motif associated with *Vcam1* regulatory regions, supporting direct transcriptional regulation of *Vcam1* by NF-κB signaling.
